# Development and validation of a novel personalized electronic patient-reported outcome measure to assess quality of life (Q-LIFE): a prospective observational study in people with Cystic Fibrosis

**DOI:** 10.1016/j.eclinm.2023.102116

**Published:** 2023-07-27

**Authors:** Danya Muilwijk, Tessa J. van Paridon, Doris C. van der Heijden, Brenda M. Faber-Bisschop, Domenique D. Zomer-van Ommen, Harry G.M. Heijerman, Cornelis K. van der Ent

**Affiliations:** a Department of Pediatric Pulmonology, Wilhelmina Children's Hospital, University Medical Center Utrecht, Lundlaan 6, 3584 EA, Utrecht, the Netherlands; b Department of Pulmonology, University Medical Center Utrecht, Heidelberglaan 100, 3584 CX, Utrecht, the Netherlands; c Dutch Cystic Fibrosis Foundation (NCFS), Dr. Albert Schweitzerweg 3a, 3744 MG, Baarn, the Netherlands

**Keywords:** Cystic Fibrosis, Quality of life, Patient-reported outcome measures, Personalized medicine

## Abstract

**Background:**

Generic and disease-specific patient-reported outcome measures (PROMs) may lack relevance and sensitivity on a patient-level in chronic diseases with differential disease expression and high individual variability, such as Cystic Fibrosis (CF). This study aimed to develop and validate a novel personalized electronic PROM (ePROM) that captures relevant aspects of quality of life in individuals with CF.

**Methods:**

The Q-Life app was developed as a short personalized ePROM to assess individual quality of life. Psychometric properties were assessed in a single-center cross-sectional study between September 2019 and September 2021 and in a prospective cohort study between September 2021 and September 2022.

**Findings:**

Combined studies included 223 participants (median age: 24 years, IQR: 19.0–32.5 years, range: 12.0–58.0 years). Internal consistency (Cronbach's alpha: 0.83–0.90) and test-retest reliability (intraclass correlation coefficient: 0.90; 95% CI: 0.65–0.92; p < 0.001) of quality of life (Q-Life) scores were strong. Q-Life scores were associated with overall Cystic Fibrosis Questionnaire-Revised (CFQ-R) scores (ρ = 0.71; p < 0.001), CFQ-R respiratory domain scores (ρ = 0.57; p < 0.001) and forced expiratory volume in 1s (ρ = 0.41; p < 0.001). Furthermore, Q-Life scores improved from 65.0 (IQR: 45.0–63.3) at baseline to 84.2 (IQR: 75.0–95.0) and 87.5 (IQR: 75.0–100.0) after 3 and 6 months of elexacaftor/tezacaftor/ivacaftor treatment (change: 20.8; 95% CI: 17.5–25.0; p < 0.001), comparable to CFQ-R respiratory domain scores (change: 22.2, 95% CI: 19.4–25.0, p < 0.001).

**Interpretation:**

The Q-Life app is a reliable, valid and sensitive personalized ePROM to measure all aspects of quality of life that really matter to individuals with Cystic Fibrosis. This patient-centered approach could provide important advantages over generic and disease-specific PROMs in the era of personalized medicine and value-based healthcare.

**Funding:**

10.13039/100000897Dutch Cystic Fibrosis Foundation, 10.13039/100016036Health-Holland.

**Research in context**

**Evidence before this study**

Patient-reported outcome measures (PROMs) play an important role in clinical trials and contribute to the transition towards a more value-based and patient-centered healthcare system. Traditional generic and disease-specific PROMs may lack relevance and sensitivity on a patient-level in chronic diseases such as Cystic Fibrosis (CF), due to heterogeneous disease manifestations and disparities in treatment options for people with different disease characteristics, leading to highly variable individual life perspectives. Personalized PROMs may better capture the broader impact of disease, new treatment modalities and healthcare on individual patients, yet such personalized tools have not been developed and validated so far.

We searched PubMed using the query “(patient-reported outcome measure [MeSH Terms]) OR (patient-reported outcome [MeSH Terms])) AND (cystic fibrosis [MeSH Terms]” for articles published up to March 6th, 2023. No language restrictions were used. Reference lists and related articles were also screened for additional relevant studies. The search identified 26 articles reporting of generic and disease-specific PROMs in CF, including two recent reviews summarizing all PROMs used in CF research and care. These reviews showed that the Cystic Fibrosis Questionnaire-Revised (CFQ-R) is by far the most commonly used and best validated disease-specific PROM, but also emphasized the urgent need for a novel, more relevant and patient-centered electronic PROM that allows for remote monitoring.

**Added value of this study**

This is the first study, to our knowledge, describing a personalized electronic PROM (Q-Life app) that is able to capture all aspects of quality of life that matter to individual patients. The app was validated in a cohort of 223 people with CF of a wide age range with different genotypes, varying disease manifestations and treatments. This personalized PROM may provide important advantages over traditional generic and disease-specific PROMs as it is short, electronic and solely focused on items that are meaningful and relevant to individual patients.

**Implications of all the available evidence**

This first validation study demonstrated the value of a personalized PROM to assess the impact of CF disease and highly effective CFTR modulator treatment on quality of life of individuals with CF. Future studies should be performed to assess external validation of the Q-Life app in different CF populations and settings and to elucidate the potential of a personalized PROM for other chronic diseases.

## Introduction

The importance of adequate patient-reported outcome measures (PROMs) that are able to capture relevant health benefits from a patient's perspective is increasingly acknowledged in medical research and healthcare. Appropriate validation, reporting and application of PROMs can support pharmaceutical labeling claims, facilitate treatment reimbursement, assist in shared-decision making and contribute to the transition towards a more value-based and patient-centered healthcare system.^1^, ^2^, ^3^, ^4^, ^5^, ^6^, ^7^ Furthermore, there is a growing need for sufficiently validated remote-monitoring tools such as electronic PROMs (ePROMs), as the digitalization in medical research and care has rapidly gained momentum since the COVID-19 pandemic.

PROMs can be defined as questionnaires that collect information on health status, as experienced and reported directly by the patient.^2^ Over the last decades, numerous generic and disease-specific PROMs have been developed, which are generally focused on symptoms, treatment satisfaction, functional status or health-related quality of life.^8^ Although disease-specific PROMs are considered to be more sensitive and reflective of patient symptoms and functioning than generic PROMs,^3^ disease-specific PROMs are still composed of a fixed list of questions related to pre-defined domains that may lack relevance and sensitivity on a patient-level. Moreover, the growing number of disease-specific PROMs hampers comparability of outcomes among patients with different diseases.

Sporadically, patient-specific outcome measures such as goal-attainment scaling have been developed and applied in different medical disciplines.^9^, ^10^, ^11^, ^12^, ^13^ In goal-attainment scaling (GAS), individual treatment goals are defined together with the patient's healthcare team, whereas scoring is performed by an independent assessor.^11^ Consequently, this method does not fulfill the criteria of a PROM, yet it has been demonstrated that individualized approaches such as GAS can be meaningful and sensitive to systematically measure the impact of treatment modalities and healthcare in a patient-centered way.^9^^,^^10^^,^^14^

Personalized approaches can be particularly useful for chronic diseases with heterogeneous clinical manifestations, such as Cystic Fibrosis (CF). CF is a rare genetic multi-system disease that causes severe symptoms and progressive functional loss of e.g. the respiratory and digestive tract. This can have a profound but varying impact on quality of life, depending on the severity, type and progression of disease manifestations as well as on available treatment options.^15^ CF could be considered as a model of other chronic diseases for which an effective new treatment was introduced recently.^16^^,^^17^

In this study, we aimed to develop a short personalized ePROM that is able to capture all important aspects of quality of life on an individual level, which we validated in people with CF (pwCF)**.**

## Methods

### Q-Life app development and features

The Q-Life app was developed in close collaboration with pwCF and parents of children with CF, who were invited and recruited by the Dutch Cystic Fibrosis Foundation (NCFS). The development process is summarized in the supplementary methods. Supplementary Figure S1 illustrates how the Q-Life app was used in this study. In the app, users can describe three to five items they find important for their personal quality of life in an open text field, and rank these items in order of importance (Supplementary Figure S1a). Each item has to be labeled with the most appropriate category, which can be selected from a pre-defined list. Subsequently, users can score for each item to what degree they currently feel limited by CF on a 5-point Likert scale, ranging from 1 (almost completely limited) to 5 (not limited), as shown in Supplementary Figure S1b. If desired, the app supports real-time visualization of results (Supplementary Figure S1c). Other features include a profile page to collect demographic variables and a brief stepwise manual. Time to complete demographics and compose the personal set of quality of life items takes approximately 5–10 min, whereas scoring only takes about 1 min. The app was downloaded from the Apple store and Google Play store. Online instruction videos are available in Dutch (https://youtu.be/986zX9Z_Cqo) and English (https://youtu.be/3dNTdeI2TYE) and can be found on YouTube by searching “Q-Life CF”. The Q-Life app and accompanying software is compliant with international data protection guidelines (ISO27001, NEN7510, ISAE3000).

### Study design, population and procedures

**Fig. 1:**
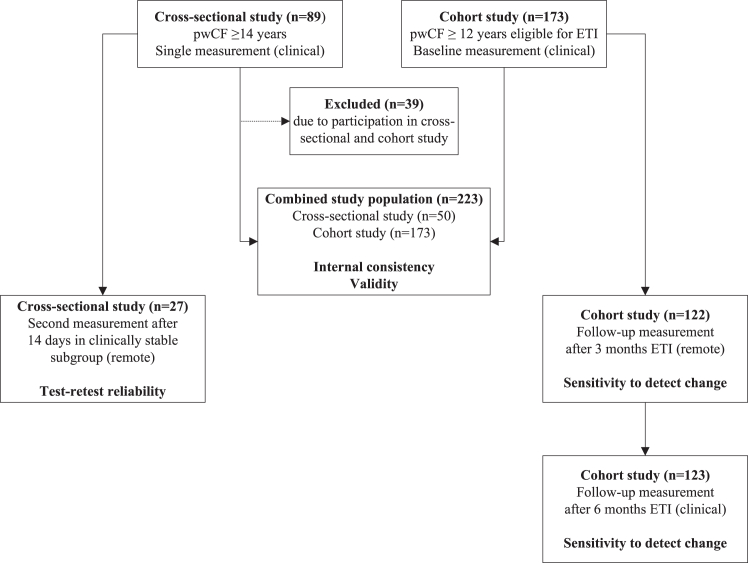
**Flowdiagram of study design and population**. Psychometric properties of the Q-Life app were assessed in a cross-sectional study including people with CF (pwCF) aged 14 years and older and in a prospective cohort study in pwCF aged 12 years and older who were eligible for elexacaftor/tezacaftor/ivacaftor (ETI). As 39 individuals with CF participated in both studies, their data collected in the cross-sectional study were excluded from the analyses in the combined study population.

This study consisted of two phases. First, we conducted a cross-sectional study in clinically stable people with a confirmed diagnosis of CF aged 14 years and older. Participants were recruited between September 2019 and September 2021, during a routine visit to the outpatient adult or pediatric CF clinic of the University Medical Center (UMC) Utrecht in the Netherlands. In this cross-sectional study, participants composed a personal set of three to five self-described quality of life items labeled with the most appropriate category and performed a single measurement to what degree they currently felt limited by CF. A clinically stable subgroup was asked to complete a second measurement after 14 days (Fig. 1). In the second phase, pwCF aged 12 years and older who were eligible for treatment with elexacaftor/tezacaftor/ivacaftor (ETI) were enrolled in a prospective observational cohort study between September 2021 and September 2022 in the UMC Utrecht. These participants were asked to describe, label and score their personal quality of life items (Q-Life items) at the baseline clinical visit prior to ETI initiation, remotely after 3 months and during a clinical visit after 6 months of treatment (Fig. 1).

Evaluation of scale reliability and validity was based on the cross-sectional study and the baseline data of the prospective cohort study, whereas sensitivity to detect change was solely derived from the cohort study (Fig. 1). Additional demographic and clinical data were collected at each study visit, including age, educational level, *Cystic Fibrosis Transmembrane Conductance Regulator protein* (CFTR) genotype, prior CFTR modulator use, lung function, expressed as Forced Expiratory Volume in 1s percentage predicted (FEV1%pred), calculated according to Global Lung Initiative guidelines^18^ and the Cystic Fibrosis Questionnaire-Revised, which is currently the reference standard of CF disease-specific quality of life.^19^ In addition, we collected the total number of pulmonary exacerbations (PEx) requiring intravenous (IV) antibiotic treatment in the year prior to the baseline visit, defined as IV-treated PEx.

### Statistical analysis

Descriptive statistics were used to summarize participant characteristics and personal Q-Life items. Per participant, overall Q-Life scores were calculated for every completed measurement in the Q-Life app. This overall Q-Life score was standardized on a 0- to 100-point scale, calculated by the sum of scores for each self-described quality of life item, expressed as percentage of the maximum possible score. To evaluate the psychometric properties of the Q-Life app, we assessed reliability and validity of Q-Life scores in the combined study population, including data of the cross-sectional study and baseline data of the cohort study participants. If pwCF participated in both studies, we only included their cohort study baseline data in the analyses (Fig. 1). Sensitivity to detect change was evaluated in the cohort study (Fig. 1). Reliability was assessed by means of internal consistency using Cronbach's α. In addition, test-retest reliability of Q-Life scores was evaluated in the clinically stable participants of the cross-sectional study who completed a second measurement in the Q-Life app after 14 days. In this subgroup (n = 27), intraclass correlation coefficients (ICC) were calculated for overall Q-Life scores (average measures), and for the separate scores of the first three personal Q-Life items (single measures) using a two-way mixed model for absolute agreement.^20^ As only a limited number of this subgroup defined a fourth (n = 14) and fifth (n = 7) personal Q-Life item, sample size was too low to calculate ICCs for these last two separate items. Content validity could only be assessed on an individual level, because the content of Q-Life items varies across participants and setting. As participants described individual Q-Life items that were important and relevant to their personal situation, the content was verified by a member of the study team during the first study visit. To assess construct validity, we calculated Spearman's correlation coefficients (Spearman's rho = ρ) of overall Q-Life scores with FEV1%pred, CFQ-R respiratory domain scores and with overall CFQ-R scores, which was calculated by the mean of the twelve CFQ-R domain scores. This overall CFQ-R score is not a standard procedure of the CFQ-R scoring, but was added in this study to provide a complimentary score that extends beyond one specific subdomain, with the aim to improve comparability with overall Q-Life scores which are derived from varying categories (i.e. domains) per participant. Subgroup analyses were conducted to estimate the impact of age and sex on the strength of these associations. In addition, we assessed the difference in median overall Q-Life scores between participants who experienced at least one IV-treated PEx and those without IV-treated PEx, between children aged 12–18 years and adults ≥18 years and between females and males (unpaired Wilcoxon's signed rank test). Similar analyses were performed for CFQ-R respiratory domain scores and overall CFQ-R scores. Finally, we assessed sensitivity to detect change by calculating the absolute change in overall Q-Life scores before and 3 and 6 months after commencement with ETI in complete cases (Paired Wilcoxon's signed rank test), in relation to the change in CFQ-R respiratory domain and overall CFQ-R scores. Absolute changes per individual Q-Life item were summarized by category. A p-value <0.05 was considered statistically significant. All hypothesis tests were two-sided. All analyses were performed in R version 4.3.0.

### Ethics statement

All participants provided written informed consent for this study, which was approved by the Institutional Review Board of the UMC Utrecht (#16-668 and #19-344).

### Role of the funding source

The Dutch Cystic Fibrosis Foundation (NCFS) recruited patient representatives who were actively involved in the development process of the Q-Life app. Furthermore, Domenique D. Zomer-van Ommen reviewed the manuscript on behalf of the NCFS. The NCFS and Health Holland had no role in the study design or in the collection, analysis and interpretation of the data. The NCFS and Health Holland also did not have access to the dataset and had no role in the decision to submit the manuscript for publication.

## Results

### Study population

In total, 89 participants enrolled in the cross-sectional study. Of this group, 27 clinically stable participants performed a second measurement in the Q-Life app after 14 days (median: 14 days, IQR: 14.0–14.5 days). The cohort study included 173 participants. As 39 individuals with CF participated in both studies, we excluded their measurements from the cross-sectional study. This resulted in a total of 223 study participants in the overall analysis (Fig. 1). The study population represented people with a wide range of age (median: 24 years, IQR: 19.0–32.5 years, range: 12.0–58.0 years), a variety of *CFTR* genotypes and prior use of different CFTR modulators (Table 1). Median overall Q-Life score at study enrollment was 66.7 (IQR: 50.0–87.5). At baseline, ceiling effects were observed in 12% of the participants who obtained the maximum overall Q-Life score of 100, compared to 4% with the maximum CFQ-R respiratory domain score of 100.

**Table 1:** Baseline characteristics.

	Combined study population (n = 223)
Cross-sectional study, no.	89
Cohort study, no.	173
Both studies, no.	39
Total included in final analysis, no.	223
CFTR genotype, no (%)	
Homozygous F508del	154 (69.1)
F508del/MF	41 (18.4)
F508del/RF	8 (3.6)
F508del/gating	6 (2.7)
F508del/unknown	6 (2.7)
MF/MF	6 (2.7)
MF/RF	1 (0.4)
MF/unknown	1 (0.4)
CFTR modulator treatment^a^, no. (%)	
None	51 (22.9)
Ivacaftor	6 (2.7)
Lumacaftor/ivacaftor	57 (25.6)
Tezacaftor/ivacaftor	92 (41.3)
Elexacaftor/tezacaftor/ivacaftor	17 (7.5)
Sex, no. (%)	
Female	108 (48.4)
Male	115 (51.6)
Level of education, no. (%)	
None	3 (1.3)
Lower/elementary school	8 (3.6)
Preparatory secondary vocational school	27 (12.1)
Secondary vocational school	63 (28.3)
Secondary school	31 (13.9)
Higher professional education	54 (24.2)
University	33 (14.8)
Missing	4 (1.8)
Age (years), median (IQR; range)	24.0 (19.0–32.5; 12.0–58.0)
Age category, no. (%)	
12–18 years	45 (20.2)
≥18 years	178 (79.8)
FEV1%pred, mean (SD; range)	71.8 (20.5; 19.0–122.0)
FEV1%pred category, no (%)	
<40%pred	14 (6.3)
40–70% pred	85 (38.1)
70–90% pred	75 (33.6)
90–110% pred	44 (19.7)
>110% pred	5 (2.3)
IV-treated PEx^b^, no. (%)	
None	169 (75.8)
One or more	54 (24.2)
BMI in adults (kg/m^2^) ≥ 18 years, mean (SD; range)	21.9 (2.7; 16.7–35.9)
BMI in adults (kg/m^2^) ≥ 18 years, category, no (%)	
<18 kg/m^2^	9 (5.1)
18–21 kg/m^2^	60 (33.7)
21–24 kg/m^2^	72 (40.4)
>24 kg/m^2^	37 (20.8)
BMI Z-score in children 12–18 years, mean (SD; range)	−0.2 (0.9; −2.0–1.8)
BMI Z-score in children 12–18 years, category, no (%)	
<−1	9 (20.0)
−1–+1	29 (64.4)
>1	7 (15.6)
Overall Q-Life score, median (IQR)	66.7 (50.0–87.5)
CFQ-R respiratory domain score, median (IQR)	72.2 (61.1–88.9)
Overall CFQ-R score^c^, median (IQR)	77.2 (66.2–85.9)

Abbreviations: BMI: body mass index; CFTR: Cystic fibrosis transmembrane conductance regulator; CFQ-R: Cystic Fibrosis Questionnaire-Revised; FEV1%pred: forced expiratory volume in 1s percentage predicted; IV: intravenous; MF: minimal function; PEx: pulmonary exacerbations; RF: residual function.

a CFTR modulator treatment at the time of study enrollment.

b IV-treated PEx in year prior to first study visit.

c The overall CFQ-R score was calculated by the mean of the twelve CFQ-R domain scores.

### Individual quality of life items

**Fig. 2:**
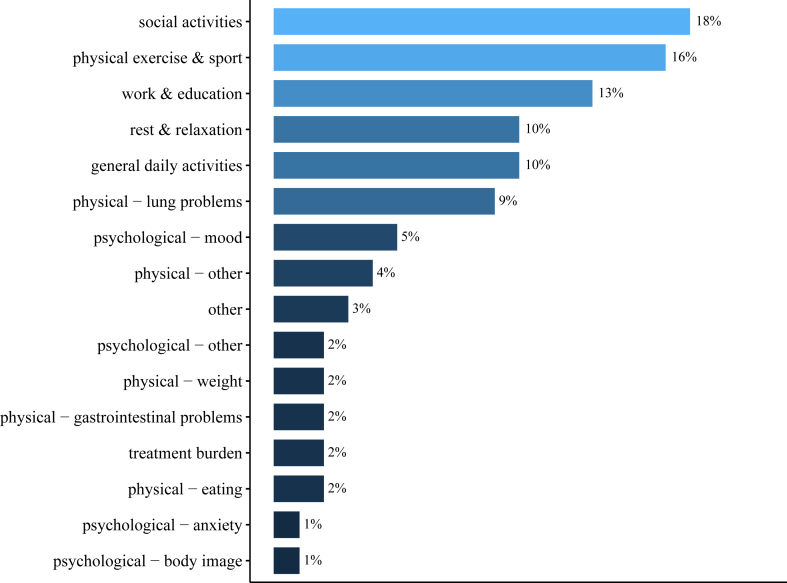
**Distribution of categories selected to label self-described quality of life items**. Study participants described a total of 858 personal Q-life items. These items had to be labeled with one of the 16 pre-defined categories that participants considered most appropriate.

Overall, 96 participants (43%) described three personal Q-Life items, whereas 65 participants (29%) and 62 participants (28%) reported four and five items, respectively. This resulted in a total of 858 self-described Q-Life items. As illustrated in Fig. 2, these items were most frequently labeled with the categories: social activities (n = 150; 18%), physical exercise and sport (n = 139; 16%), work and education (n = 114; 13%), general daily activities (n = 89; 10%), rest and relaxation (n = 82; 10%) and physical – lung problems (n = 78; 9%). Examples of self-described Q-Life items are provided for each category in Supplementary Table S1.

### Reliability

Internal consistency of individual Q-Life scores was high, based on Cronbach's α of 0.83 when at least three personal Q-Life items were described (n = 223). Consistency was slightly higher when assessed in those who described at least four (n = 127) or five items (n = 62), with Cronbach's α of 0.87 and 0.90, respectively. The subgroup of the cross-sectional study (n = 27) showed an excellent stability of overall Q-Life scores after 14 days (ICC: 0.90; 95% CI: 0.65–0.92; p < 0.001). This was consistent when assessed separately for the first three self-described items, according to ICCs of 0.73–0.82 (Table 2).

**Table 2:** Test-retest reliability of Q-Life scores (n = 27).

Q-Life scores	ICC^a^	95% CI	p-value
Overall score	0.90	0.78–0.96	<0.001
Item 1	0.73	0.49–0.86	<0.001
Item 2	0.81	0.62–0.91	<0.001
Item 3	0.73	0.48–0.87	<0.001

Abbreviation: ICC: intraclass correlation coefficient.

a ICCs were calculated for overall Q-Life scores and for the separate scores of the first three self-described items at baseline and after 14 days in a clinically stable subgroup of participants.

### Validity

**Fig. 3:**
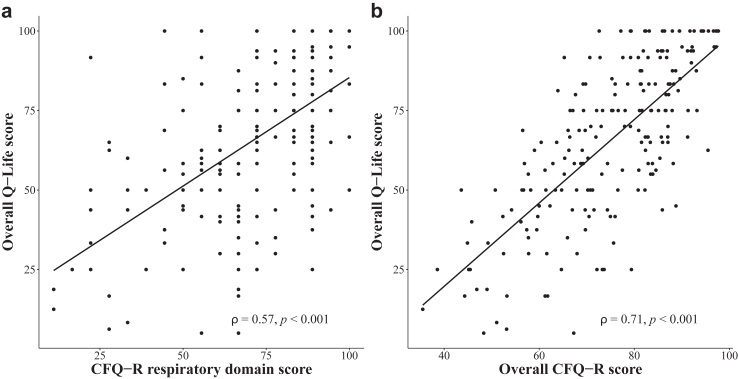
**Association of overall Q-Life scores with CFQ-R scores**. Overall Q-Life scores were moderately associated with Cystic Fibrosis Questionnaire-Revised (CFQ-R) respiratory domain scores (a) and with overall CFQ-R scores, calculated by the mean of the twelve CFQ-R domain scores (b). ρ = Spearman's correlation coefficient (black line).

Construct validity of Q-Life scores was assessed in multiple ways. First, overall Q-Life scores were associated with FEV1%pred (ρ = 0.41, p < 0.001), which indicates that participants with a better lung function reported higher Q-Life scores. In addition, overall Q-Life scores were positively associated with CFQ-R respiratory domain scores (ρ = 0.57, p < 0.001) and overall CFQ-R scores (ρ = 0.71, p < 0.001; Fig. 3). We did not observe a substantial impact of age or sex on the strength of these associations (Supplementary Figure S2). Furthermore, overall Q-Life scores were able to capture differences in CF disease severity, as pwCF who did not experience any IV-treated PEx had higher overall Q-Life scores compared to those who experienced at least one IV-treated PEx in the year prior to study participation (median difference: 16.3, 95% CI: 6.7–25.0, p < 0.001). The association between overall Q-Life scores and IV-treated PEx is shown in Supplementary Table S2 and Supplementary Figure S3. In addition, children with CF aged 12–18 years reported higher overall Q-Life scores than adults ≥18 years (median difference: 18.3, 95% CI: 10.0–25.0, p < 0.001). Overall Q-Life scores did not significantly differ between females and males (difference in median: −1.8, 95% CI: −8.3–5.0, p = 0.70). Similar characteristics were observed in our data for CFQ-R respiratory domain scores and overall CFQ-R scores (Supplementary Table S3).

### Sensitivity to detect change

**Fig. 4:**
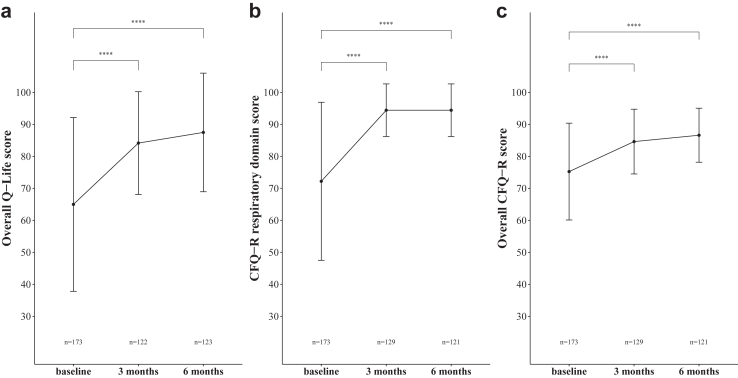
**Sensitivity to detect change of overall Q-Life and CFQ-R scores**. Median overall Q-Life scores significantly changed from baseline after 3 and 6 months of treatment with elexacaftor/tezacaftor/ivacaftor (a). The magnitude of change was comparable to the median change in CFQ-R respiratory domain scores (b), and higher than the median change in overall CFQ-R scores (c). Error bars represent median absolute deviation. Significance level p < 0.001 = ∗∗∗∗.

After 3 months of treatment with ETI, 122/173 (71%) of the participants completed a second Q-Life measurement remotely (Fig. 1), whereas 129/173 (75%) also completed the CFQ-R. In total, 145/173 (84%) of the participants returned to the clinical follow-up visit after 6 months of ETI. Of this subgroup, 123/145 participants completed a Q-Life measurement (Fig. 1) and 121/145 completed the CFQ-R, indicating that Q-Life and CFQ-R data were missing for 51/173 (29%) and 44/173 (25%) participants after 3 months of ETI, as well as for 50/173 (29%) and 51/173 (29%) participants after 6 months of treatment, respectively. Supplementary Table S4 shows a comparison of baseline characteristics between participants who did and did not complete a follow-up Q-Life measurement after 3 and 6 months. Median overall Q-Life score improved from 65.0 (IQR: 45.0–63.3) at baseline to 84.2 (IQR: 75.0–95.0) after 3 months and subsequently to 87.5 (IQR: 75.0–100.0) after 6 months, with a difference in median of 20.8 (95% CI: 17.5–25.0, p < 0.001 in paired samples; Fig. 4a). The magnitude of change was comparable to the change in CFQ-R respiratory domain score (difference in median: 22.2, 95% CI: 19.4–25.0, p < 0.001; Fig. 4b), which improved from 72.2 (IQR: 55.6–88.9) at baseline to 94.4 (IQR: 83.3–100.0) after 3 months and remained 94.4 (IQR: 83.3–100.0) after 6 months. Both changes were considerably higher than the change in overall CFQ-R score, which increased from 75.3 (IQR: 65.3–85.5) at baseline to 84.6 (IQR: 77.4–90.9) and 86.6 (IQR: 79.1–91.7), after 3 and 6 months, respectively (difference in median: 10.0, 95% CI: 7.9–12.1, p < 0.001; Fig. 4c). As illustrated in Fig. 4, the CFQ-R seemed to have reached a ceiling after 3 months of treatment with ETI. Q-Life scores demonstrated a comparable scale-responsiveness, but showed slightly lower absolute values after 3 and 6 months of treatment and a larger individual variance, suggesting that its ceiling may not have been reached. Median changes per self-described Q-Life item are summarized by category in Supplementary Table S5.

## Discussion

The Q-Life app is a short personalized ePROM, developed in co-creation with patients and validated to capture quality of life on an individual level in 223 pwCF ranging in age from 12 to 58 years.

Reliability, validity and sensitivity to detect change of personal quality of life scores measured with the Q-Life app were good to excellent. These psychometric properties are at least comparable or slightly better than reported for the CFQ-R, which is the most widely used disease-specific PROM in CF.^21^^,^^22^ The respiratory symptom subscale of the CFQ-R has been validated most extensively^19^^,^^23^ and is still the main focus of important CF-related clinical trials to demonstrate the impact of new treatments on quality of life.^16^^,^^17^^,^^24^, ^25^, ^26^

Interestingly, however, our results illustrated that individuals with CF did not frequently consider respiratory symptoms as important or relevant to their quality of life, as personal Q-Life items related to lung problems were only described in 9% of total. Although respiratory symptoms are a hallmark of CF,^15^ these findings suggest that assessment of disease-specific symptoms may not be not sufficient to capture quality of life for most pwCF. Furthermore, it emphasizes the added value of a patient-centered personalized PROM like the Q-Life app, which is sensitive to track changes in other quality of life domains that are important and relevant for individuals.

Different types of PROMs require different validation approaches, which is acknowledged by regulatory authorities.^2^ As personal Q-Life items are self-described by individual participants and not pre-defined, validation of the content of a personalized tool deviates from the regular validation process of standardized generic and disease-specific PROMs.^2^ The Q-Life app intends to measure the same general construct of personal quality of life, but the content of the personal quality of life items is variable between participants and will inherently vary across different settings. This indicates that content validity can only be assessed on an individual level. Even though the content of individual items is derived directly from the participant, verification will be necessary to ensure content validity per individual and setting.

Internal consistency and test-retest reliability of Q-Life scores were high to excellent, even with a limited set of three to five self-described quality of life items with a content that varied per individual. These reliability measures were stronger than observed in the CFQ-R validation study.^19^ Consistency seemed to increase with an increasing number of personal Q-Life items, although this might be influenced by the lower sample size of the groups who selected four and five personal items. In terms of consistency and relevance, three to five personal items seemed sufficient to capture all relevant aspects of quality of life for the majority of study participants, but the most optimal number of personal items might vary in different settings and should be further researched. Criterion validity and construct validity were demonstrated by associations of overall Q-Life scores with CFQ-R scores, which were regarded as reference standard to measure the concept quality of life in pwCF, as well as with measures of disease severity such as FEV1%pred, IV-treated PEx and age. The association and discriminative capacity of Q-Life scores and CFQ-R scores with measures of disease severity were comparable in our data and slightly better than previously reported for the CFQ-R,^19^ substantiating validity of the Q-Life app. The association of Q-Life scores with FEV1%pred seems to be slightly lower than the association between the CFQ-R scores with FEV1%pred, although these correlations fall within the same range. These findings might be explained by the fact that Q-Life scores were only partly based on respiratory symptoms in 9% of participants, whereas respiratory symptoms have a more prominent role in the CFQ-R. This also supports the hypothesis that quality of life in general is only partly dependent on lung function or respiratory symptoms.

The ceiling effects at baseline may be explained by the liberal method of describing personal quality of life items, as participants were asked to describe items that were most important and relevant for their personal situation, which does not necessarily mean that these aspects are also affected by CF. As ceiling effects cause an increased skewness of the score distribution and subsequently an underestimation of the mean, we only used median scores in the analysis of this study. Ceiling effects generally reduce sensitivity to detect change, yet the cohort study showed that the Q-Life app was still sensitive to detect a group-level change in median overall Q-Life scores, at least when highly effective CFTR modulator therapy is initiated. The responsiveness of overall Q-Life scores was comparable to median CFQ-R respiratory domain scores and much higher than the changes in median overall CFQ-R scores in our data as well as in the non-respiratory CFQ-R domains in the phase 3 ETI trials.^27^ Post-ETI, absolute median overall Q-Life scores were slightly lower than CFQ-R respiratory domain scores and individual variability was substantially higher. As illustrated by our data, sensitivity to detect change may diminish in pwCF who are becoming less symptomatic, e.g. in those already using highly effective CFTR modulators, but also in children or in those with mild disease manifestations. Further research is warranted to examine and compare the ceiling effects and sensitivity to detect change of the Q-Life app and CFQ-R in these specific CF populations.

Several socioeconomic and clinical factors are associated with health-related quality of life of adolescents and adults with CF. Physical symptoms including lung function decline and pulmonary exacerbations as well as mental symptoms such as anxiety and depression usually have the broadest impact.^28^^,^^29^ The current treatment landscape of CF, however, has led to profound changes in life perspectives of pwCF who are eligible for highly effective CFTR modulator therapy, which is in contrast with the urgent unmet need for personalized therapies for those who carry rare CFTR mutations that cannot be treated with these modulators. Therefore, the heterogeneous nature of CF disease manifestations and advancing but disparate treatment options ask for a more flexible, patient-centered approach to adequately capture the impact of CF disease, treatment modalities and healthcare on individuals.

The Q-Life app is a unique tool aimed to measure what really matters to individual patients, as it contains a short, easy to use personalized list of important and relevant items, which takes little time to be composed and scored. The personalized nature and relevance, efficiency, sensitivity and flexibility of the Q-Life app could provide advantages over the relatively large and burdensome set of questionnaires that are currently used in CF, but additional studies will be needed to assess whether the Q-Life app has the potential to replace at least some of these traditional questionnaires in the future. In addition, ePROMs have general advantages over paper-based PROMs in terms of feasibility, utility, accuracy, acceptance and response rates, and are more easily integrated into electronic research data capture systems and medical records.^30^ In the cohort study, we observed similar response rates for the remote visit and the clinical visit, suggesting that remote monitoring could provide a suitable opportunity to maintain contact with individuals with CF who do not need to be frequently monitored in-hospital. The relatively limited time to complete a measurement in the Q-Life app might allow for more frequent data entry (e.g. weekly or bi-weekly), but the flexibility of the Q-Life app supports accommodation to the most optimal frequency in different settings such as trials or healthcare, and may also be tailored to individual preferences.

There are several important limitations to this study. In this observational study, we demonstrated the use of a patient-specific PROM to assess the impact of CF disease and highly effective CFTR modulator treatment on quality of life of individuals with CF. Additional studies are warranted for further development and external validation of this personalized ePROM in different CF populations including ethnically minoritized individuals, children and parents or caregivers, in different countries and settings such as clinical trials and healthcare, and in the context of e.g. different treatment modalities or life events. In addition, the minimal clinical important difference should be assessed in future studies.^31^ Further research may also elucidate the value of a personalized ePROM such as the Q-Life app in other chronic diseases. The time period of this observational study was also an important limitation, as the largest part was conducted during the COVID-19 pandemic. Although all participants were explicitly instructed to score the impact of CF on their quality of life, we could not rule out that the COVID-19 pandemic, including the intermittent social distancing measures, may have had an impact on the study results. We were not able to include adolescents with CF in the panel of patient representatives who were involved in the development of the Q-Life app due to lack of availability, indicating that this part of the target population was underrepresented in the initial development phase. In addition, not all members of the CF multidisciplinary team were involved in the core development team, indicating that potentially valuable input might have been missed. Furthermore, a follow-up measurement in the cohort study was missing in 29% of participants. This could have over- or underestimated the sensitivity to detect change of the Q-Life app, although it was still comparable to the CFQ-R respiratory domain scores.

In conclusion, this first validation study showed that the Q-Life app is a reliable, valid and sensitive personalized ePROM to assess all aspects of quality of life that really matter to individuals with CF.

## Contributors

Concept and design: D. Muilwijk, B.M. Faber-Bisschop, C.K. van der Ent. Acquisition, analysis or interpretation of data: D. Muilwijk, T.J. van Paridon, D.C. van der Heijden, H.G.M. Heijerman, C.K. van der Ent. Drafting of the manuscript: D. Muilwijk. Critical revision of the manuscript for important intellectual content: D. Muilwijk, T.J. van Paridon, D.C. van der Heijden, B.M. Faber-Bisschop, D.D. Zomer-van Ommen, H.G.M. Heijerman, C.K. van der Ent. Statistical analysis: D. Muilwijk. Obtained funding: D. Muilwijk, C.K. van der Ent. Administrative, technical or material support: D.D. Zomer-van Ommen. Supervision: C.K. van der Ent. Data access: D. Muilwijk, T.J. van Paridon, D.C. van der Heijden, H.G.M. Heijerman, C.K. van der Ent. Data verification: D. Muilwijk, T.J. van Paridon, D.C. van der Heijden. Decision to submit the manuscript: D. Muilwijk, H.G.M. Heijerman, C.K. van der Ent.

## Data sharing statement

The study data and metadata will be made available upon request to the corresponding author.

## Declaration of interests

C.K. van der Ent reported a grant (CFOS 2022) from The Dutch Cystic Fibrosis Foundation (money to institution), outside of the submitted work. No other disclosures were reported.
