# Supplementary material for: Development and validation of a novel personalized electronic patient-reported outcome measure to assess quality of life (Q-LIFE): a prospective observational study in people with Cystic Fibrosis
Source: eClinicalMedicine. 2023 Jul 27;62:102116. doi: 10.1016/j.eclinm.2023.102116 (PMC10404867; doi:10.1016/j.eclinm.2023.102116)
Supplement: Supplementary Figures and Tables [file mmc1.docx]

**Supplementary Material**

**Content**

1. Supplementary methods. Q-Life app development
2. Supplementary table 1. Examples of self-described quality of life items per category
3. Supplementary table 2. Logistic regression model estimates of IV-treated PEx and overall Q-Life scores
4. Supplementary table 3. Construct validity of Q-Life scores compared to CFQ-R scores
5. Supplementary table 4. Comparison of baseline characteristics between subgroups with complete and missing Q-Life measurements
6. Supplementary table 5. Q-Life scores per self-described item grouped by category
7. Supplementary figure 1. Overview of Q-Life app
8. Supplementary figure 2. Associations of overall Q-Life scores with FEV1% Pred and CFQ-R scores by age and sex
9. Supplementary figure 3. Association of IV-treated PEx with Q-Life scores

**Supplementary methods:** Q-Life app development

The Q-Life app was developed as a personalized electronic patient-reported outcome measure intended to assess quality of life on an individual level in people with Cystic Fibrosis (pwCF). The development team consisted of a panel of three adults with Cystic Fibrosis (CF) and two parents of children with CF who accepted the invitation of the Dutch Cystic Fibrosis Foundation (NCFS) to participate as patient representatives in this project. In addition, one coordinating clinical researcher, two physicians with CF expertise (one adult respiratory physician and one pediatrician) and two software developers were part of this core development team. To ensure a central role of pwCF in this project, one of the CF panel members was involved in the entire development process. The research coordinator of the NCFS had an advisory role throughout the development process.

In the first development phase, the impact of CF on individual quality of life and the desirable properties of an individualized measurement tool were discussed during a focus group meeting and an individual interview with all 5 panel members using a standardized template of questions. The focus group discussion and individual interviews led to the decision to create an app in which pwCF can enter self-described items they consider important and relevant for their personal quality of life in an open text field. The aim was to create an efficient and relevant tool, keeping the number of items as low as possible and including only those aspects that were considered important and relevant for someone’s individual quality of life. The panel considered three to five items as sufficient for this purpose. The instructions about how to describe and formulate the personal Q-Life items were also derived from this focus group and from the individual interviews. Personal items were considered as the primary and most important aspect of the app. The categories that were used to label these self-described items were intended to have a supportive role, so they were solely created to facilitate standardization and validation against the Cystic Fibrosis Questionnaire-Revised (CFQ-R). For this reason, we used the domains of the CFQ-R as a starting point. During the focus group meeting, categories were extended and renamed to improve understanding of the categories, as it is important for users to understand the categories to be able to label personal items with a most appropriate one.

After the development of the Q-Life app, pre-testing was conducted with the five panel members. Based on one cycle of cognitive interviews, all categories were retained. The categories body image and treatment burden were renamed to improve understanding of this category. In addition, the instructions about how to describe personal quality of life items were slightly modified. Furthermore, we added the instruction to complete the following sentence: “I find it important that I …”, aimed to facilitate standardization of the self-described personal quality of life items. Survey length was not modified. Most testers indicated that the ranking of the personal items in order of importance was difficult or deemed irrelevant. Therefore, we decided to drop the ranking as a compulsory part of the app in these studies, but retained this as optional feature. All other modifications based on the pre-testing were related to the design and functioning of the app and aimed to improve clarity, user-friendliness and bug fixing. The first version of the app was also discussed and tested by the CF multidisciplinary team, who provided additional input regarding the content, clarity and design of the app. After completing the second development phase, the app was considered ready for use.

**Supplementary table 1:** Examples of self-described quality of life items per category

| **Example – I find it important that I…** ^a^ | **Category** |
| --- | --- |
| Can take care of myself, my animals and my household independently | General daily activities |
| Can work fulltime | Work and education |
| Can dance like the others, without getting extremely tired | Physical exercise and sport |
| Find a good balance between exertion and relaxation | Relaxation and rest |
| Have enough energy to spend time with friends | Social activities |
| Have few respiratory infections | Physical – lung problems |
| Experience few abdominal complaints, a calm bowel | Physical – gastrointestinal problems |
| Can enjoy food | Physical – eating |
| Maintain a stable weight | Physical – weight |
| Am in a more consistent shape, so I can rely on how much energy I’ll have tomorrow | Physical – other |
| Feel happy | Psychological – mood |
| Learn to accept the uncertainty of the future | Psychological – anxiety |
| Maintain a positive image of my body | Psychological – body image |
| Can feel mentally at peace | Psychological – other |
| Have more time for other activities instead of having to undergo long intensive nebulization therapy | Treatment burden |
| Can participate in society | Other |

^a^ Examples of self-described quality of life items, labelled with categories that could be selected from a pre-defined list. Participants were instructed to describe their personal items by completing the following sentence: “I find it important that I…”.

**Supplementary table 2**: Logistic regression model estimates of IV-treated PEx and overall Q-Life scores

|  | **Odds ratio** | **95% confidence interval** | **p-value** |
| --- | --- | --- | --- |
| Intercept | 1·40 | 0·61–3·19 | 0·423 |
| Overall Q-Life score | 0·98 | 0·96–0·99 | <0·001* |

Abbreviations: IV: intravenous; PEx: pulmonary exacerbations.
Interpretation: the odds of IV-treated PEx decreased with increasing Q-Life scores.
*Significance level p<0·05.

**Supplementary table 3:** Construct validity of Q-Life scores compared to CFQ-R scores

|  | **Q-Life scores (95% CI)** | **CFQ-R respiratory subdomain score (95% CI)** | **CFQ-R overall scores (95% CI)** |
| --- | --- | --- | --- |
| Association with FEV1%pred | 0·41*** | 0·49*** | 0·50*** |
| Difference between groups with and without IV-treated PEx ^a^ | 16.3 (6.7–25.0)*** | 16·7 (11·1–22·2)*** | 9·6 (5·1–14·3)*** |
| Difference between children 12-18 years and adults >18 years ^b^ | 18·3 (10·0–25·0)*** | 11·1 (5·6–16·7)*** | 8·3 (3·9–12·9)*** |
| Difference between females and males | -1·8 (-8·3–5·0) ^ns^ | -5·6 (-11·1–0·0) ^ns^ | 0·9 (-2·9–4·6) ^ns^ |

Abbreviations: CFQ-R: Cystic Fibrosis Questionnaire-Revised; FEV1%pred: forced expiratory volume in 1s percent predicted; IV: intravenous; PEx: pulmonary exacerbations.

^a^ Difference in median indicates higher quality of life scores in group without IV-treated PEx compared to group with at least 1 IV-treated PEx in year prior to first study visit.

^b^ Difference in median indicates higher quality of life scores in children compared to adults.

*** Significance level p<0·001. ns= not significant.

**Supplementary table 4**: Comparison of baseline characteristics between subgroups with complete and missing Q-Life measurements after 3 and 6 months of treatment with elexacaftor/tezacaftor/ivacaftor (ETI)

|  | **Follow-up Q-Life measurement after 3 months ETI** | | **Follow-up Q-Life measurement after 6 months ETI** | |
| --- | --- | --- | --- | --- |
| **Baseline characteristics** | **Complete (n=122)** | **Missing (n=51)** | **Complete (n=123)** | **Missing (n=50)** |
| CFTR genotype, no (%) |  |  |  |  |
| Homozygous F508del | 97 (79·5) | 39 (76·5) | 100 (81·3) | 36 (72·0) |
| F508del/MF | 23 (18·9) | 6 (11·7) | 22 (17·9) | 7 (14·0) |
| F508del/RF | - | 2 (3·9) | - | 2 (4·0) |
| F508del/gating | 1 (0·8) | 1 (2·0) | - | 2 (4·0) |
| F508del/unknown | 1 (0·8) | 3 (5·9) | 1 (0·8) | 3 (6·0) |
| CFTR modulator treatment ^a^, no. (%) |  |  |  |  |
| None | 24 (19·7) | 11 (21·5) | 23 (18·7) | 12 (24·0) |
| Ivacaftor | 1 (0·8) | 1 (2·0) | - | 2 (4·0) |
| Lumacaftor/ivacaftor | 37 (30·3) | 18 (35·3) | 39 (31·7) | 16 (32·0) |
| Tezacaftor/ivacaftor | 60 (49·2) | 21 (41·2) | 61 (49·6) | 20 (40·0) |
| Sex, no. (%) |  |  |  |  |
| Female | 57 (46·7) | 25 (49·0) | 60 (48·8) | 22 (44·0) |
| Male | 65 (53·3) | 26 (51·0) | 63 (51·2) | 28 (56·0) |
| Level of education, no. (%) |  |  |  |  |
| None | 2 (1·7) | 1 (2·0) | 2 (1·6) | 1 (2·0) |
| Primary/elementary school | 4 (3·3) | 1 (2·0) | 4 (3·3) | 1 (2·0) |
| Preparatory secondary vocational school | 12 (9·8) | 10 (19·6) | 12 (9·8) | 10 (20·0) |
| Secondary vocational school | 36 (29·5) | 17 (33·3) | 39 (31·7) | 14 (28·0) |
| Secondary school | 16 (13·1) | 9 (17·6) | 17 (13·8) | 8 (16·0) |
| Higher professional education | 33 (27·0) | 7 (13·7) | 34 (27·6) | 6 (12·0) |
| University | 17 (13·9) | 5 (9·8) | 14 (11·4) | 8 (16·0) |
| Missing | 2 (1·7) | 1 (2·0) | 1 (0·8) | 2 (4·0) |
| Age (years), median (IQR) | 24·0 (19·0–30·8) | 24·0 (19·0–31·0) | 24·0 (19·5–29·0) | 23·0 (17·0–34·0) |
| FEV1%pred, mean (SD) | 75·0 (20·3) | 74·0 (17·6) | 73·2 (20·1) | 75·8 (18·4) |
| IV-treated PEx^b^, no. (%) |  |  |  |  |
| None | 96 (78·7) | 41 (80·4) | 91 (74·0) | 46 (92·0) |
| One or more | 26 (21·3) | 10 (19·6) | 32 (26·0) | 4 (8·0) |
| BMI in adults (kg/m²) ≥ 18 years, mean (SD) | 21·7 (2·4) | 21·2 (2·5) | 21·6 (2·3) | 21·5 (2·7) |
| BMI Z-score in children 12-18 years, mean (SD) | -0·2 (1·0) | 0·0 (0·7) | -0·2 (1·0) | -0·1 (0·8) |
| Q-Life score, median (IQR) | 66·7 (50·0–83·3) | 58·3 (42·7–75·0) | 66·7 (47·5–83·3) | 59·1 (43·8–91·3) |
| CFQ-R respiratory domain score, median (IQR) | 72·2 (59·7–88·9) | 72·2 (50·0–83·3) | 72·2 (55·6–83·3) | 72·2 (55·6–88·9) |
| Overall CFQ-R score^c^, median (IQR) | 77·3 (65·3–85·7) | 73·3 (63·7–83·9) | 75·3 (64·9–85·0) | 74·4 (65·7–86·2) |

Abbreviations: BMI: body mass index; CFTR: Cystic fibrosis transmembrane conductance regulator; CFQ-R: Cystic Fibrosis Questionnaire-Revised; ETI: elexacaftor/tezacaftor/ivacaftor; FEV1%pred: forced expiratory volume in 1s percentage predicted; IV: intravenous; MF: minimal function; PEx: pulmonary exacerbations; RF: residual function.

^a^ CFTR modulator treatment at the time of study enrollment.

^b^ IV-treated PEx in year prior to first study visit.

^c^ The overall CFQ-R score was calculated by the mean of the twelve CFQ-R domain scores.

**Supplementary table 5:** Q-Life scores per self-described item grouped by category

| **Category** | **Q-Life scores at baseline** | | **Q-Life scores after 6 months ETI** | |
| --- | --- | --- | --- | --- |
|  | **n** | **Median (IQR)** | **n** | **Median (IQR)** |
| General daily activities | 68 | 4·0 (3·0–5·0) | 45 | 4·0 (4·0–5·0) |
| Work and education | 91 | 4·0 (3·0–5·0) | 68 | 5·0 (4·0–5·0) |
| Physical exercise and sport | 112 | 4·0 (3·0–4·0) | 82 | 5·0 (4·0–5·0) |
| Relaxation and rest | 65 | 4·0 (3·0–5·0) | 43 | 5·0 (4·0–5·0) |
| Social activities | 116 | 4·0 (3·0–5·0) | 83 | 5·0 (4·0–5·0) |
| Physical – lung problems | 65 | 3·0 (2·0–4·0) | 47 | 5·0 (4·0–5·0) |
| Physical – gastrointestinal problems | 16 | 3·0 (2·0–3·0) | 12 | 3·5 (3·0–4·0) |
| Physical – eating | 19 | 3·0 (3·0–4·0) | 13 | 4·0 (3·0–5·0) |
| Physical – weight | 12 | 3·0 (2·0–3·3) | 6 | 3·5 (3·0–4·8) |
| Physical – other | 27 | 3·0 (3·0–4·0) | 16 | 4·0 (4·0–5·0) |
| Psychological – mood | 33 | 3·0 (3·0–4·0) | 24 | 4·0 (2·0–4·3) |
| Psychological – anxiety | 7 | 2·0 (2·0–2·5) | 6 | 3·5 (2·3–4·0) |
| Psychological – body image | 11 | 3·0 (2·0–4·0) | 8 | 4·0 (3·0–4·0) |
| Psychological – other | 9 | 3·0 (1·0–5·0) | 9 | 5·0 (3·0–5·0) |
| Treatment burden | 12 | 3·0 (3·0–4·0) | 10 | 5·0 (4·0–5·0) |
| Other | 14 | 3·5 (2·3–4·8) | 9 | 4·0 (3·0–5·0) |

Abbreviations: ETI: elexacaftor/tezacaftor/ivacaftor; n: number of self-described items in cohort study for which a score was completed at baseline and after 6 months of treatment with ETI.

**Supplementary figure 1:** Overview of Q-Life app


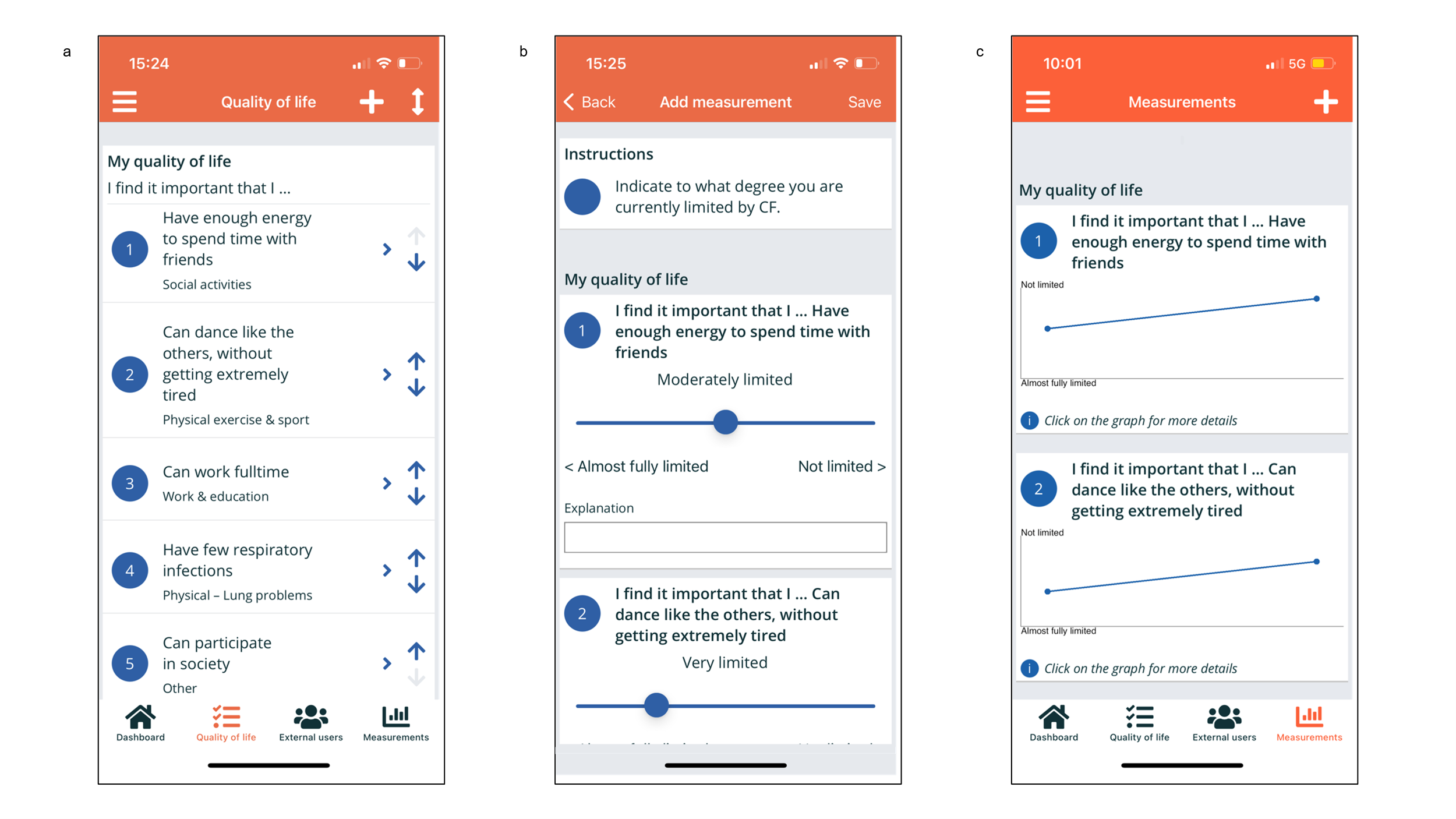


a) Step 1: example of a personal set of self-described Q-Life items, labelled with the most appropriate category. Personal items can be ranked by using the arrows on the right. b) Step 2: for each item, users can score to what degree they currently feel limited by CF on a 5-point Likert scale: almost fully limited (1 point), very limited (2 points), moderately limited (3 points), minimally limited (4 points) or not limited (5 points). c) Graphical display of results.

**Supplementary figure 2**: Associations of overall Q-Life scores with FEV1%pred and CFQ-R scores in subgroups


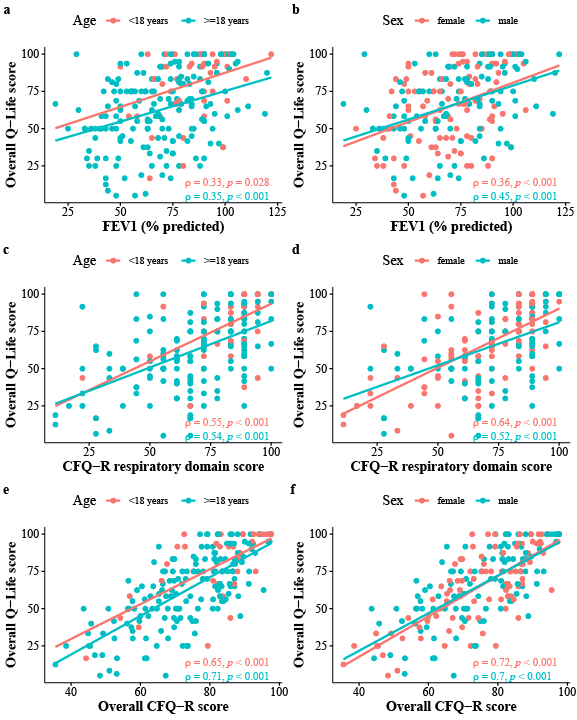


The strength of the associations between overall Q-Life scores and FEV1%pred, CFQ-R respiratory domain scores and overall CFQ-R scores did not substantially differ between age groups (a,c,e) or between females and males (b,d,f). ρ = Spearman’s correlation coefficient.

**Supplementary figure 3**: Association of IV-treated PEx in the year prior to study participation with overall Q-Life scores


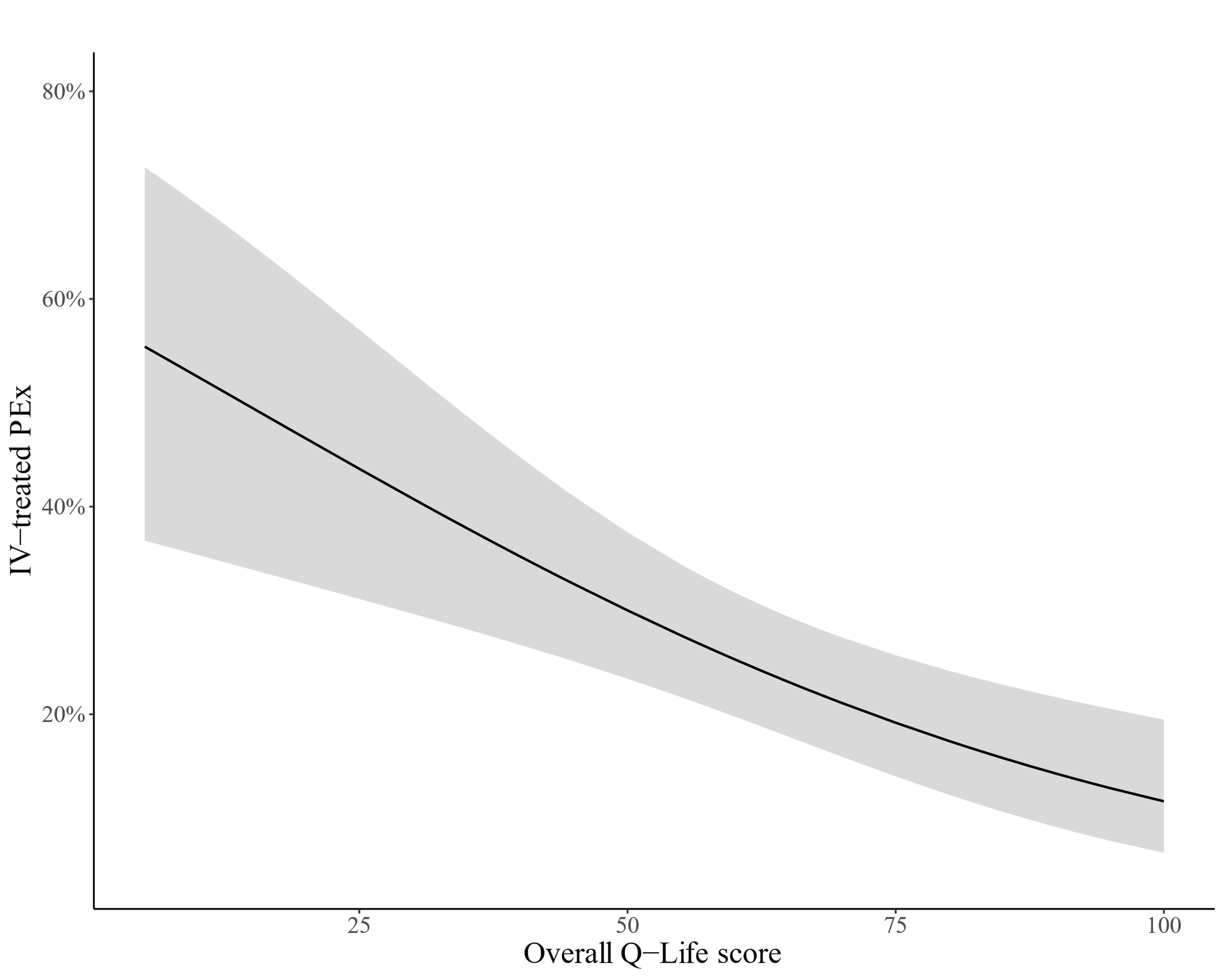


The probability of experiencing a pulmonary exacerbation treated with intravenous antibiotics (IV-treated PEx) decreases with an increasing Q-Life score. The black line represents the average model estimates derived from Supplementary Table 2. The grey ribbon represents the 95% confidence interval.
